# A case report: axillary ALK-positive anaplastic large-cell lymphoma mimicking an infectious abscess in an adolescent

**DOI:** 10.3389/fonc.2026.1915361

**Published:** 2026-10-02

**Authors:** Zhanwei Wang, Liuhong Yuan, Xudong Guan, Xiaohui Liu, Yuanting Song, Kun Tao, Tao Dai

**Affiliations:** 1 Department of Wound Healing, Tongji Hospital, Tongji University School of Medicine, Shanghai, China; 2 Department of Pathology, Tongji Hospital, Tongji University School of Medicine, Shanghai, China

**Keywords:** anaplastic large-cell lymphoma (ALCL), axillary abscess-like lesion, biopsy, case report, soft-tissue infection, wound management

## Abstract

**Background:**

Abscess-like presentations are common in surgical practice, but malignant soft-tissue and lymphoid disorders can occasionally imitate infection and delay definitive treatment. Anaplastic large-cell lymphoma (ALCL) is a CD30-positive T-cell lymphoma that may involve skin, lymph nodes, or soft tissue and can show extensive necrosis, inflammatory change, and systemic symptoms.

**Case presentation:**

A 17-year-old female student presented with a 3-week history of progressive painful erythema and swelling of the left axilla after ineffective intravenous antibiotic treatment elsewhere. On admission, the lesion was large, indurated, tender, and clinically suspicious for a soft-tissue infection with possible abscess formation. Incision and drainage yielded only scant purulent fluid but abundant friable nodular tissue, and cultures were negative. Persistent fever, local progression, lymphadenopathy, and poor response to antimicrobial therapy prompted multidisciplinary reassessment. Histopathology of the solid tissue demonstrated large pleomorphic lymphoid cells, and immunohistochemistry showed strong CD30 and ALK expression, establishing ALK-positive ALCL. The patient was transferred to hematology and received initial cytoreductive therapy with brentuximab vedotin and short-course zeprumetostat, after which the local tumor burden decreased and delayed wound closure was performed. She subsequently received five cycles of brentuximab vedotin plus CHP and chidamide maintenance after complete remission.

**Conclusion:**

A presumed abscess that yields little pus but contains solid friable tissue should not be managed as infection alone. In refractory, culture-negative, or biologically discordant soft-tissue lesions, early biopsy of the cavity wall and solid components may help reduce diagnostic delay and redirect care toward malignancy-specific treatment.

## Introduction

Axillary abscesses and cellulitis-like soft-tissue infections are familiar problems for surgeons. The usual clinical pathway is pragmatic: source control, drainage when indicated, culture, and antimicrobial therapy. This approach is appropriate for most patients, but it can become hazardous when the apparent abscess is actually a malignant or lymphoproliferative process with secondary necrosis. In that setting, repeated drainage and escalating antibiotics may treat the appearance of disease while the underlying diagnosis continues to progress.

Anaplastic large-cell lymphoma (ALCL) is a mature T-cell lymphoma characterized by strong CD30 expression and distinctive anaplastic cytology (1, 2). Systemic ALK-positive ALCL is defined by ALK rearrangement and ALK protein expression, occurs more commonly in children, adolescents, and young adults, and may involve nodal and extranodal sites, including skin and soft tissue (3). Although ALK-positive disease generally has a more favorable prognosis than ALK-negative ALCL, diagnostic delay may occur when extranodal disease presents with inflammatory signs, necrosis, fever, or local tenderness that mimic infection.

The novelty of this case lies not in the diagnosis of ALK-positive ALCL itself, but in its presentation as a refractory axillary abscess-like lesion that created a surgical diagnostic challenge. The key diagnostic clue emerged intraoperatively: a large clinically inflamed lesion yielded only scant purulent fluid but contained abundant friable solid tissue. Similar diagnostic pitfalls have been reported in ALCL and in other malignancies presenting as presumed abscesses (4–6). This case therefore supports consideration of early tissue sampling when clinical, radiological, microbiological, or operative findings are discordant with uncomplicated pyogenic infection.

## Case description

A 17-year-old female high-school student presented to the Department of Wound Healing with a 3-week history of progressive redness, swelling, and pain in the left axilla. Before admission, she had received intravenous antibiotics at a local hospital for presumed soft-tissue infection, but the lesion enlarged and became increasingly painful. She had fever on admission, with a body temperature of 38.4 degrees Celsius.

Physical examination showed a diffuse, indurated, erythematous area measuring approximately 18 x 18 cm, involving the left axilla, anterior chest wall, and medial upper arm (**Figure 1A**). The skin was warm, tense, and markedly tender. Laboratory testing showed hemoglobin 123 g/L, white blood cell count 6.89 x 10^9/L, red blood cell count 4.62 x 10^12/L, and platelet count 307 x 10^9/L. C-reactive protein was 5.7 mg/L. Procalcitonin was 0.437 ng/mL. The notable abnormality was eosinophilia, with eosinophils accounting for 19% of the differential count. Ultrasonography demonstrated a mixed-echogenicity mass with irregular hypoechoic areas and was interpreted as a mixed mass with possible abscess.

**Figure 1:**
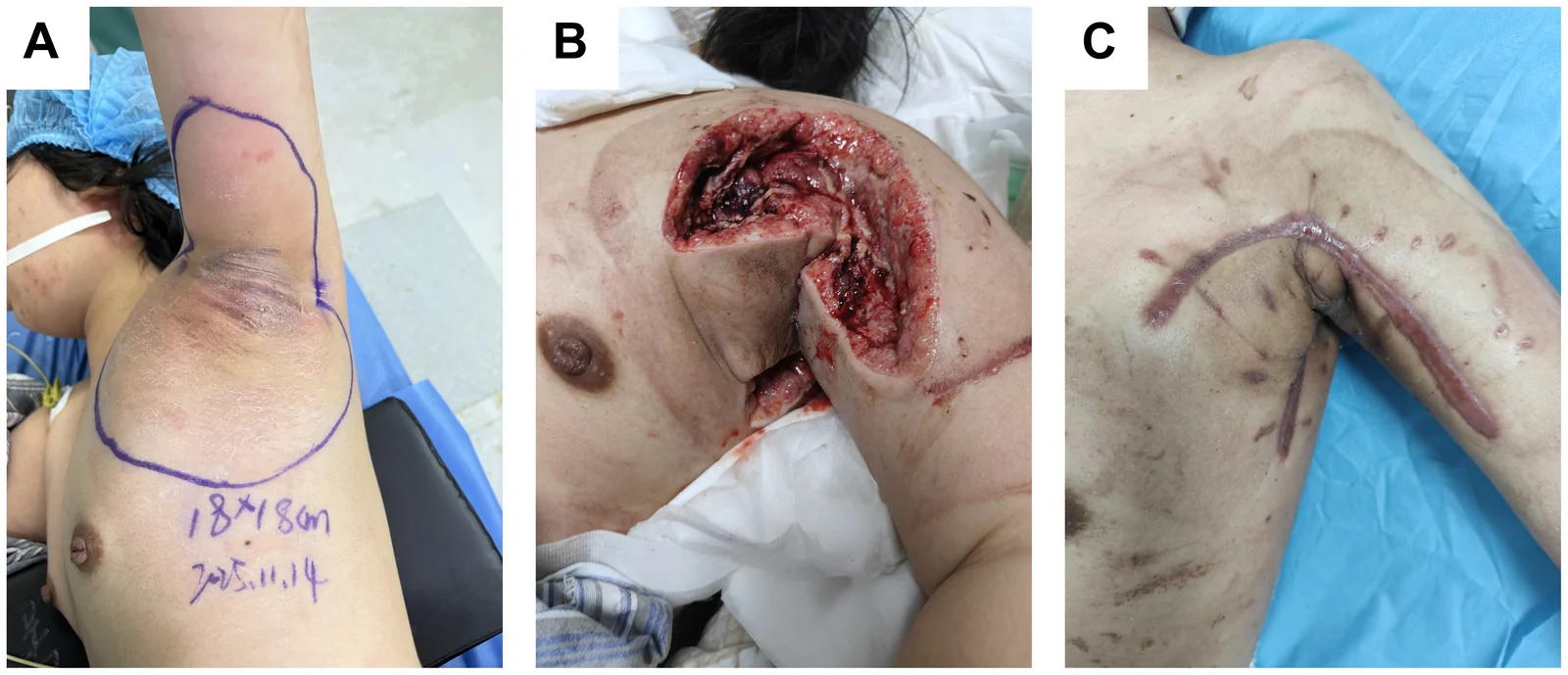
Clinical evolution of the axillary lesion. **(A)** Initial presentation showing a diffuse, indurated, erythematous mass measuring approximately 18 × 18 cm and involving the left axilla, anterior chest wall, and medial upper arm. **(B)** Postoperative refractory phase showing wound dehiscence, swollen and indurated margins, necrotic tissue in the wound bed, and absence of significant purulent discharge. **(C)** Final local outcome after lymphoma-directed therapy and delayed surgical closure, showing complete wound healing.

Because the lesion was painful, tense, progressive, and refractory to prior antibiotics, incision and drainage were performed on the day of admission. The operative findings were not typical of an uncomplicated pyogenic abscess. Only a small amount of purulent fluid was evacuated; most of the cavity consisted of friable solid nodular tissue. The cavity was decompressed and debrided, pus was sent for microbiological testing, and the solid granular tissue was submitted for histopathological examination.

After surgery, the patient received empirical cefuroxime and supportive wound care. Her local symptoms improved only slightly, and the wound later showed necrosis and poor healing rather than the expected improvement after drainage. Inflammatory indices subsequently rose, fever persisted, and on November 16, 2025, antibiotics were escalated to cefoperazone-sulbactam. On November 17, 2025, erythema over the anterior chest wall had worsened and the upper limb remained tense. Chest computed tomography showed multiple nodular and mass-like low-density shadows in the left axilla, blurred fat spaces near the axilla and chest wall, and multiple enlarged lymph nodes.

A second operation was performed on November 18, 2025. The incision was extended from the anterior chest wall to the upper arm and explored to the subfascial plane. Nodular masses were again identified, with only a small amount of purulent secretion between tissue planes. Debridement, decompression, and vacuum sealing drainage were performed. Postoperative antimicrobial and anti-edema treatment did not produce the expected response (**Figure 1B**). On November 21, 2025, antimicrobial therapy was escalated to meropenem 0.5g every 8 h, but intermittent fever continued and local improvement remained limited. Repeat CT on November 22, 2025, showed postoperative changes, local inflammatory change with small gas collections, multiple enlarged lymph nodes, and marked swelling of the adjacent chest-wall subcutaneous tissue.

## Diagnostic assessment

The diagnosis shifted after multidisciplinary review of three discordant features: the large abscess-like lesion produced little pus; repeated cultures were negative; and adequate drainage plus broad-spectrum antibiotics did not control fever or local progression. The marked eosinophilia was reinterpreted as a possible paraneoplastic clue rather than a minor inflammatory finding.

Histopathological examination of the excised solid tissue showed diffuse infiltration by large atypical lymphoid cells with abundant cytoplasm and pleomorphic nuclei, including hallmark-like kidney- or horseshoe-shaped nuclei (**Figure 2A**). Immunohistochemistry demonstrated diffuse strong CD30 expression and ALK positivity (**Figures 2B, C**). These findings established ALK-positive anaplastic large-cell lymphoma. The disease was classified clinically as ALK-positive ALCL, stage II B group according to the source clinical record.

**Figure 2:**
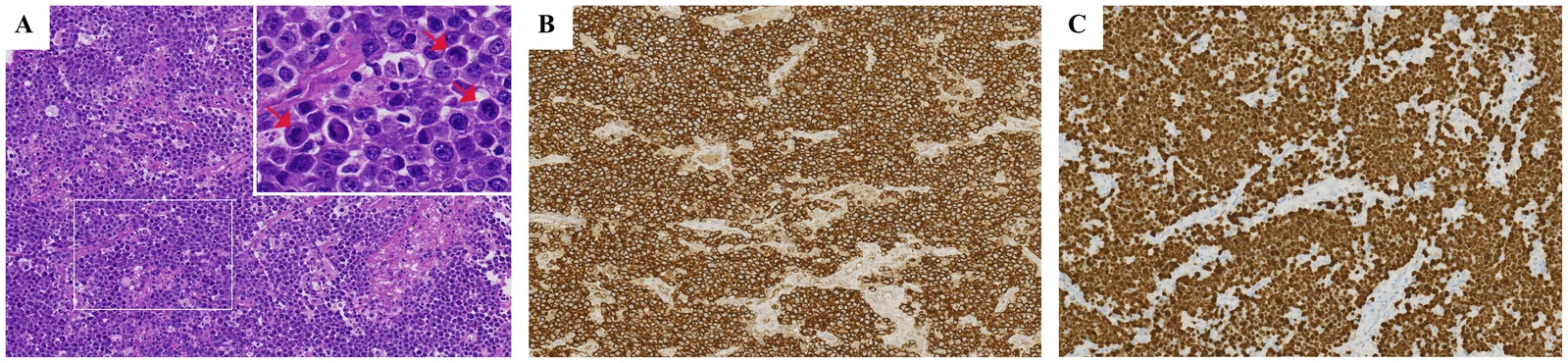
Histopathological and immunohistochemical findings. **(A)** Hematoxylin and eosin staining showing diffuse infiltration of large atypical lymphoid cells; high-power inset highlights large pleomorphic cells with abundant cytoplasm and kidney- or horseshoe-shaped nuclei. **(B)** CD30 immunohistochemistry showing diffuse strong membranous/Golgi-region tumor-cell staining. **(C)** ALK immunohistochemistry showing diffuse tumor-cell positivity, supporting ALK-positive ALCL.

## Therapeutic intervention

After the pathological diagnosis was established on November 24, 2025, the patient was transferred to the Department of Hematology for systemic lymphoma-directed therapy. Initial cytoreductive treatment consisted of brentuximab vedotin 100 mg intravenously on November 28, 2025, followed by zeprumetostat, an oral EZH2 inhibitor, at 350 mg every 12 h from December 1, 2025 for one course. After adequate regression of the local tumor burden and improvement of the wound bed, delayed surgical closure was performed on December 9, 2025. The postoperative course was uneventful, the patient was discharged on December 16, 2025, and the wound subsequently achieved complete healing (**Figure 1C**).

After discharge, the patient received five cycles of CD30-targeted therapy combined with a CHP regimen on December 31, 2025, January 22, 2026, February 10, 2026, March 3, 2026, and March 25, 2026. Each cycle consisted of brentuximab vedotin 100 mg on day 1, cyclophosphamide 1180 mg on day 1, epirubicin 110 mg on day 1, and prednisone 95 mg on days 1-5. Follow-up assessment on April 24, 2026 indicated complete remission, after which brentuximab vedotin plus CHP was discontinued and chidamide maintenance therapy was started. Follow-up examinations on July 13, 2026 and September 5, 2026 showed no abnormal findings. At the latest follow-up, the patient remained on chidamide maintenance therapy, with good wound healing and no evidence of local recurrence. The key clinical events are summarized in **Table 1**.

**Table 1:** Timeline of clinical events.

Date	Event	Clinical significance
Before Nov 14, 2025	Three weeks of progressive left axillary erythema, swelling, and pain	Refractory course despite presumed infection management
Nov 14, 2025	Presentation to wound-healing department; fever 38.4 °C; 18 × 18 cm axillary/anterior chest-wall lesion; eosinophils 19%; ultrasound suggested possible abscess	Infection considered, but eosinophilia and discordant inflammatory profile were atypical
Nov 14, 2025	Incision and drainage; scant pus but abundant friable nodular tissue; culture and biopsy obtained	Key intraoperative red flag: abscess-like mass with solid tissue predominance
Nov 16, 2025	Antibiotics escalated to cefoperazone-sulbactam	Persistent fever and local progression
Nov 17, 2025	CT showed multiple nodular/mass-like soft-tissue shadows, blurred fat planes, and lymphadenopathy	Imaging no longer favored a simple drained abscess
Nov 18, 2025	Second debridement and decompression with vacuum sealing drainage	Persistent nodular tissue and small amount of purulent secretion
Nov 21, 2025	Antibiotics escalated to meropenem	Ongoing fever despite broad-spectrum therapy
Nov 22, 2025	Repeat CT showed postoperative changes, lymphadenopathy, and marked subcutaneous swelling	Continued refractory course
Nov 24, 2025	Pathology diagnosed ALK-positive ALCL; multidisciplinary discussion; transfer to hematology	Definitive diagnosis and change in treatment strategy
Late Nov-Dec 2025	Brentuximab vedotin- and zeprumetostat-based therapy; daily wound care	Rapid regression of swelling and fever
Dec 9, 2025	Delayed wound closure	Local disease controlled enough for reconstruction
Dec 16, 2025	Discharge	Wound healing trajectory established
Dec 24, 2025	Complete wound healing; regular hematology follow-up	Favorable local surgical outcome after lymphoma-directed therapy

## Discussion

This case illustrates a high-risk diagnostic pitfall: malignant lymphoma can present to surgical services as an abscess-like emergency. The initial decision to operate was clinically reasonable. The lesion was painful, tense, progressive, febrile, and sonographically compatible with a possible abscess; prior antibiotics had failed. The central lesson is not that drainage should have been avoided, but that drainage should be converted into diagnostic surgery when operative findings contradict the working diagnosis.

The hematologic diagnosis changed the interpretation of the surgical findings. ALK-positive ALCL is a CD30-positive mature T-cell lymphoma defined by ALK rearrangement or ALK protein expression, and this biologic profile has direct diagnostic and therapeutic implications (1–3). In the present case, strong CD30 and ALK expression confirmed that the friable solid tissue encountered during drainage was not merely inflamed granulation tissue, but lymphoma involving the axillary soft tissue. This explains why source control and broad-spectrum antibiotics produced only limited improvement, whereas lymphoma-directed therapy was followed by rapid local regression and wound-bed recovery.

The strongest signal was the mismatch between mass effect and purulent yield. A large, inflamed, tense axillary lesion would usually be expected to produce a corresponding collection if it were a simple pyogenic abscess. Instead, the cavity contained abundant friable nodular tissue. This finding supports consideration of early tissue sampling, including biopsy of the cavity wall and nodular components when feasible, during the first procedure. Such an early tissue-sampling approach may prevent repeated debridement and antibiotic escalation when infection is not the primary process; **Figure 3** summarizes this experience as a conceptual framework to support clinical recognition and early tissue sampling in similar abscess-like soft-tissue lesions.

**Figure 3:**
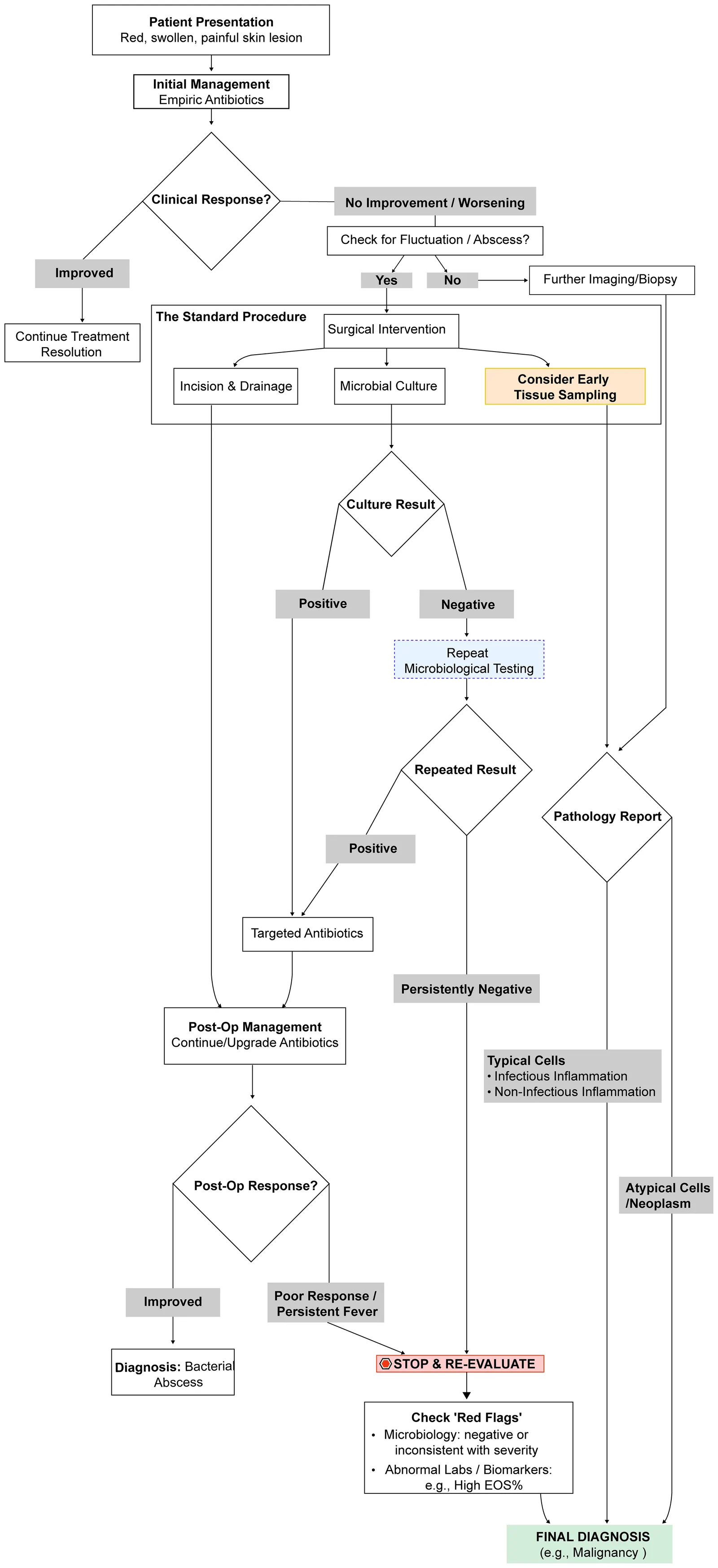
Conceptual framework for early tissue sampling in refractory abscess-like soft-tissue lesions. The framework summarizes the diagnostic warning signs observed in this case, including scant purulent drainage, friable solid tissue, negative cultures, poor response to antimicrobial therapy, lymphadenopathy, and unexplained eosinophilia. It is intended to support clinical awareness and decision-making in similar cases.

The second signal was the culture-negative and treatment-refractory course. Negative culture alone does not exclude infection, particularly after prior antibiotics. However, persistent fever, local progression, CT evidence of coalescing soft-tissue nodules, lymphadenopathy, and minimal response to broad antimicrobial therapy together weakened the infection-only explanation. In retrospect, these features justified early multidisciplinary reassessment.

The third signal was eosinophilia. Eosinophilia has many causes and should not be overinterpreted in isolation. In this case, however, eosinophils accounted for 19% of leukocytes in a patient whose white blood cell count and C-reactive protein level were not markedly elevated. Lymphoproliferative disorders are recognized causes of hypereosinophilia, and IL-5-related eosinophilic infiltration has been described in ALCL (7, 8). The eosinophilia therefore became meaningful when combined with sterile drainage, solid tissue, lymphadenopathy, and antibiotic failure.

The differential diagnosis of refractory abscess-like lesions should remain broad. In addition to ALCL, malignant causes include other lymphomas and soft-tissue sarcomas such as rhabdomyosarcoma, which may initially be treated as presumed abscesses (4–6). Inflammatory mimics, including pyoderma gangrenosum and hidradenitis suppurativa, may also present with painful, recurrent, culture-negative, or treatment-refractory lesions and may require dermatologic or multidisciplinary evaluation (9, 10). The present case adds to this broader clinical context by emphasizing an operative decision point: when an abscess-like lesion contains solid friable tissue, yields little pus, or behaves atypically after drainage and antimicrobial therapy, tissue sampling can redirect management toward the correct diagnosis.

The treatment strategy was based on the tumor phenotype and the need for rapid control of bulky local disease. Strong CD30 expression supported the use of brentuximab vedotin. In ECHELON-2, brentuximab vedotin combined with CHP improved outcomes compared with CHOP in previously untreated CD30-positive peripheral T-cell lymphomas, particularly systemic ALCL (11). Zeprumetostat was used as short-course individualized therapy under hematology supervision; as an oral EZH2 inhibitor, SHR2554/zeprumetostat has shown antitumor activity in mature lymphoid neoplasms, including peripheral T-cell lymphoma, in early-phase studies (12). In this case, the initial brentuximab vedotin and zeprumetostat-based phase was followed by five cycles of brentuximab vedotin plus CHP, complete remission on April 24, 2026, and chidamide maintenance, with no abnormal findings on follow-up through September 5, 2026.

This report has limitations. It describes a single patient, so the proposed early tissue-sampling approach should be interpreted as a suggested diagnostic strategy rather than a validated algorithm. Although follow-up through September 5, 2026 showed complete remission and no local wound recurrence, longer observation is still needed to assess durable oncologic control. The available materials did not include detailed molecular testing results, complete imaging-based staging data, bone marrow findings, or serial quantitative measurements of tumor burden. Nevertheless, the case provides a clear surgical lesson: when surgery for a presumed abscess reveals a biologically discordant lesion, the surgeon has a critical opportunity to secure tissue diagnosis.

## Conclusion

ALK-positive ALCL can mimic a severe axillary soft-tissue infection. In refractory or culture-negative abscess-like lesions, especially when the operative cavity contains solid friable tissue and purulence is scant, surgeons should consider histological sampling during initial operative management or early reassessment. Early biopsy of the cavity wall and nodular components can shorten diagnostic delay, prevent repeated ineffective procedures, and accelerate referral for malignancy-specific therapy.

## Data Availability

All data relevant to this case report are included in the article. Additional de-identified clinical information may be made available by the corresponding authors upon reasonable request, subject to patient privacy and institutional requirements.
