# Correction: A case report: axillary ALK-positive anaplastic large-cell lymphoma mimicking an infectious abscess in an adolescent

**DOI:** 10.3389/fonc.2026.2036098

**Published:** 2026-10-30

**Authors:** Zhanwei Wang, Liuhong Yuan, Xudong Guan, Xiaohui Liu, Yuanting Song, Kun Tao, Tao Dai

**Affiliations:** 1 Department of Wound Healing, Tongji Hospital, Tongji University School of Medicine, Shanghai, China; 2 Department of Pathology, Tongji Hospital, Tongji University School of Medicine, Shanghai, China

**Keywords:** anaplastic large-cell lymphoma (ALCL), axillary abscess-like lesion, biopsy, case report, soft-tissue infection, wound management

In the published article, there was an error in the **Funding** statement. The grant number was incorrectly stated as “GJPY2319”. The correct grant number is “RCQD2309”. The corrected **Funding** statement appears below:

“The author(s) declared that financial support was received for this work and/or its publication. This work was supported by Tongji Hospital, Tongji University School of Medicine (grant no. RCQD2309).”

The original version of this article has been updated.

